# Psoralen-ultraviolet A treatment with Psoralen-ultraviolet B therapy in the treatment of psoriasis

**DOI:** 10.12669/pjms.293.2622

**Published:** 2013

**Authors:** Sadaf Ahmed Asim, Sitwat Ahmed, Najam us-Sehar

**Affiliations:** 1 Dr. Sadaf Ahmed Asim, Assistant Professor of Dermatology, Dow International Medical College, Dow University Hospital (Ojha),; 2 Dr. Sitwat Ahmed, Consultant Dermatologist, Patel Hospital and Plastic Surgery and General Hospital,; 3 Dr. Najam-us-Saher, Consultant Dermatologist, Zainab Panjwani Memorial Hospital, Karachi, Pakistan.

**Keywords:** Psoriasis, Photochemotherapy, Ultraviolet radiation, Psoralen, UVB

## Abstract

***Objective:*** To compare the conventional psoralen-ultraviolet A treatment with psoralen-ultraviolet B therapy in the treatment of psoriasis.

***Methodology:*** We studied 50 patients of plaque type psoriasis who were selected to receive either conventional psoralen-ultraviolet A or psoralen-ultraviolet B treatment.

***Results:*** There was no significant difference between the two treatment groups in the number of patients whose skin cleared of psoriasis or the number of exposures required for clearance. Profile of side effects and disease status was also similar after three months of follow up.

***Conclusion: ***Psoralen-ultraviolet B treatment is as effective as conventional psoralen-ultraviolet A in the treatment of psoriasis. Further long term studies are needed to assess the safety of psoralen-ultraviolet B.

## INTRODUCTION

Psoriasis is a common, chronic skin disease which affects approximately 2% of the population.^1^ The disease is usually manifested as raised, well-demarcated,erythematous oval plaques with adherent silvery scales.^2^ It has many clinical variants but the most common presentation is termed psoriasis vulgaris, which affects approximately 85-90% of all patients with the disease.^3^

Many therapeutic modalities are available for the treatment of Psoriasis both topical and systemic including salicylic acid, corticosteroids, tar, dithranol and ultraviolet B (UVB), and systemic agents like photochemotherapyretinoids, methotrexate and cyclosporine.^4^ The choice of therapy is determined by many factors, like age, sex, site, severity and duration of disease and general health of the patient.

Photochemotherapy is a unique therapeutic tool, which involves combined use of a drug and ultraviolet radiations (UVR).The drug given for this purpose has the potential to sensitize the tissues to radiation so that only lower doses of radiation are sufficient to induce beneficial effects.^5^

Psoralens are a group of photosensitizing drugs which are ineffective when used alone, but in combination with UVR provide an effective tool for the treatment of psoriasis and a number of other photoresponsive skin disorders e.g. vitiligo and mycosis fungoides.^6^

Ultraviolet radiation (UVA) was first introduced scientifically by Mofti AM about 53 years ago for the treatment of vitiligo.^6^ The pharmacology of psoralens was first described in 1954^7^ and the acronym PUVA was coined by Parrish et al in 1974^8^, who also reported successful treatment of psoriasis with PUVA. In addition to UVA, UVB can also be used either alone or in combination with psoralens. Nowadays, a narrowband UVB is utilized for the same purpose which has shown greater therapeutic efficacy with minimal side effects as compared to broadband UVB.^9^

The present study was conducted to assess the efficacy, safety and tolerability of PUVA (Psoralen-ultraviolet A) and to compare the effects of PUVA and PUVB (Psoralen-ultraviolet B), a new mode of therapy in the treatment of psoriasis vulgaris.

## METHODOLOGY

This comparative interventional study was conducted in the Dermatology department of Civil Hospital Karachi, from 1^st^ April 2005 to March 31^st^, 2008. This is a dissertation based study, approval of which was taken from Research Training and Monitoring Cell, CPSP. Adult patients of both sexes having psoriasis vulgaris diagnosed clinically were included in the study. The severity of psoriasis was assessed on the basis of Psoriasis area and severity index (PASI) which is measured by assessing the erythema, in duration and desquamation in lesions. Findings of clinical history, general examination, skin examination and laboratory tests (complete blood count, liver function tests, serum creatinine) were recorded on a specially designed proforma.

Patients were divided into two groups, Group A & Group B, each group having 25 patients. Group A received PUVA and Group B received narrowband PUVB. Treatment was given twice per week for 8 weeks. Patients were exposed to UVA or UVB, two hours after intake of 8-MOP(10 mg) tablet in a dose of 0.6mg/kg body weight. The initial dose of UVA was given according to our skin type that is at least 3J/cm2. The dose was increased by 40% of the previous dose every time until erythema was achieved. The last dose before erythema was continued till clearance in 16 sessions.

The initial dose of UVB was started with 0.9 J/cm2 and the dose was gradually incremented by 10% of the previous dose until erythema was reached. The last dose was used till clearance. All the patients were advised to wear U-V blocking glasses during exposure to ultraviolet radiation. The photochemotherapy unit used in this study was an upright cabinet variety of PUVA combilight treatment cubicle, the patient being treated from all sides in a standing position. The cubicle was 4 sided without a roof and 24 UVA-F85/100w tubes were mounted side by side.

Patients were followed up for three months with only emollients to be used during this time, to assess the disease status or any side effects. PASI was calculated before & after treatment for each patient & the difference was observed. All cases of photosensitivity disorders, hepatic, renal, ophthalmic or cardiovascular diseases were excluded from the study.

## RESULTS

A total of 50 patients, 36(72%) male and 14(28%) female were included in the study. The age range was between 21-50 years. In both groups there were 18 males & 7 females. Demographic and clinical parameters of patients at baseline is presented in (Table-I). As regards skin type, 6 patients had skin type V followed by type IV in 0 patients. Three patients had type III and one patient had type II skin. The area of distribution & PASI score for both males & females is given in (Table-II).

Clearance was found in 23 (92%) out of 25 in PUVA and 20(80%) in PUVB. There was no statistically significant difference between both groups (p value <0.5) Two patients in PUVA group and five patients in PUVB group had mild improvement and were declared resistant. Psoriasis of the limbs were found to be more resistant compared to trunk and needed more sessions for clearance. (Table-III)

Table-IV describes the side effects of both groups. Pigmentation was the most commonly observed side effect seen in 32% of patients followed by nausea, vomiting, pruritis and headache. Erythroderma, blister and claustrophobia were the rare side effects. 

## DISCUSSION

Our results showed that the effects of PUVA and PUVB are comparable in terms of efficacy and safety as 23 out of 25 patients of PUVA and 20 of PUVB cleared. These results are comparable to the study done by De Berkeret al.^10^

Another study done by Tahir R, included forty patients of chronic plaque psoriasis and compared the effects of PUVA or UVB and found that treatment with PUVA resulted in clearance of psoriasis in significantly greater number of patients (85%) compared to UVB (65%) with fewer number of exposures.^11^ They concluded that PUVA was more effective treatment of psoriasis compared to narrow band UVB phototherapy.

**Table-I:** Demographic and clinical parameters of patients

*Variable*	*PUVA* *N=25*	*PUVB* *N=25*
Age range (years)	21-48	22-50
Mean age (years)	33	34
Sex (male/female)	18/7	18/7
Duration of disease(years)	2-20	1-21
Mean duration(years)	10.5	11
Extent of skin involvement (%)	25-70	30-70

**Table-II:** Comparison between results of PUVA and PUVB therapy

	*PUVA* *N=25*	*PUVB* *N=25*
Number of patients cleared	23(92%)	20(80%)
Number of treatments required for clearance	15	16
Average duration of treatment (weeks)	8	8

**Table-III:** Distribution of lesions & PASI score in PUVA & PUVB groups

*Area of distribution*	*Male* *n(%)*	*Female* *n(%)*	*Total*
Trunk	31(62%)	8(16%)	39
Upper limbs	36(72%)	14(28%)	50
Lower limbs	36(72%)	14(28%)	50
*PASI Score*			
30-40	19(76%)	8(32%)	27
41-50	7(28%)	4(16%)	11
51-60	8(32%)	1(4%)	9
61-69	2(8%)	1(4%)	3

**Table-IV:** Comparison between side effects of PUVA and PUVB therapy

*Side effect*	*PUVA(n=25)* *n(%)*	*PUVB (n=25)* *n(%)*
Erythema	7(28)	6(24)
Pruritis	8(32)	7(28)
Nausea, vomiting	7(28)	6(24)
Fever & malaise	4(16)	3(12)
Headache	3(12)	4(16)
Blister	1(4)	0(0)
Claustrophobia	1(4)	0(0)

Phototherapy (UVB) is indicated for patients with generalized plaque, guttate psoriasis, or palmoplantar psoriasis who have not responded adequately to conventional topical therapies. Since UVB is easier to administer and does not involve an oral photosensitizing medication, this form of phototherapy is often selected before psoralen photochemotherapy (PUVA).^12^

The results of Sakuntabhai et al study in the treatment of psoriasis also show the superiority of the combined therapy of psoralen plus ultraviolet B over that of UVB alone. They reported a clearance rate of 88% compared to our 80% with the use of same therapy.^13^ Similar results were reported by Khurshid et al, who achieved 77.7% clearance rate with the use of UVB compared to our 80%.^14^

The study done by Shamsuddin et al compare the efficacy and safety of psoralen UVB and UVB alone therapy in the treatment of psoriasis. They included 50 patients of plaque type psoriasis and found a slightly higher rate of clearance (84%) with PUVB than UVB alone.^15^

Another study by Bari et al compared the effects of PUVA and UVB therapy in moderate plaque psoriasis in 50 patients and concluded that both forms of treatment are effective in moderate plaque psoriasis, however UVB phototherapy should be the first choice because of lesser short term adverse reactions.^16^ In contrast some studies found PUVA to be more effective treatment for psoriasis than narrow band UVB phototherapy.^11^

Erythema was the most commonly observed side effect in our patients, a finding previously reported in the literature.^10^^,^^12^^,^^13^ Nausea & vomiting was seen in 60% of our patients which is a psoralen related side effect^10^^,^^11^^,^^14^^,^^15^ and was seen with equal frequency in PUVA & PUVB groups. Fever and malaise were other side effects which were similar in frequency in both the groups.^14^^,^^15^Pruritis was another commonly observed finding, however it may not be solely related to ultraviolet therapy as the frequency was similar in both groups. The most common cause of pruritis is dryness of the skin and it responds to lubricants. Erythema is the most common acute side effect of PUVA therapy.

It is well known that erythema from PUVA is delayed by 48 to 72 hours or longer in case of severe reaction compared to that from UVB, which usually peaks within 12 hours. In a large study, the incidence of erythema accompanying PUVA therapy was 32.3%.^16^ Our results were in agreement with other international and local studies.^10^^,^^13^^,^^14^^.^^17^ Although there were no serious short term adverse reactions seen in our study, there is an established risk of developing cutaneous malignancies with prolong use of PUVA. There is no such risk involved in UVB phototherapy. The combination of phototherapy with psoralen or newer biologic agents are now considered a preferred treatment of psoriasis.^18^

## CONCLUSION

Psoralen UVB treatment is as effective as conventional PUVA in the treatment of plaque type psoriasis. Further long term studies are needed to assess the safety of psoralen UVB.
